# Quantification of Cigarette Smoke Particle Deposition *In Vitro* Using a Triplicate Quartz Crystal Microbalance Exposure Chamber

**DOI:** 10.1155/2013/685074

**Published:** 2012-12-26

**Authors:** Jason Adamson, David Thorne, John McAughey, Deborah Dillon, Clive Meredith

**Affiliations:** British American Tobacco, Group R&D, Regents Park Road, Southampton SO15 8TL, UK

## Abstract

There are a variety of smoke exposure systems available to the tobacco industry and respiratory toxicology research groups, each with their own way of diluting/delivering smoke to cell cultures. Thus a simple technique to measure dose *in vitro* needs to be utilised. Dosimetry—assessment of dose—is a key element in linking the biological effects of smoke generated by various exposure systems. Microbalance technology is presented as a dosimetry tool and a way of measuring whole smoke dose. Described here is a new tool to quantify diluted smoke particulate deposition *in vitro*. The triplicate quartz crystal microbalance (QCM) chamber measured real-time deposition of smoke at a range of dilutions 1 : 5–1 : 400 (smoke : air). Mass was read in triplicate by 3 identical QCMs installed into one *in vitro* exposure chamber, each in the location in which a cell culture would be exposed to smoke at the air-liquid interface. This resulted in quantification of deposited particulate matter in the range 0.21–28.00 **μ**g/cm^2^. Results demonstrated that the QCM could discriminate mass between dilutions and was able to give information of regional deposition where cell cultures would usually be exposed within the chamber. Our aim is to use the QCM to support the preclinical (*in vitro*) evaluation of tobacco products.

## 1. Introduction

Modelling human disease processes *in vitro* is important for our understanding of the risks associated with human exposure to known and unknown inhaled chemicals or toxicants. These *in vitro* models can be used for mechanistic-based research and/or to assess potential harm of consumer goods, such as household products, cosmetics, or tobacco products. Although it can be argued that *in vitro* models have limitations in human physiological relevance, it is believed that these models have potential to reduce the financial and ethical burden on *in vivo* animal testing.

Looking specifically at the toxicological assessment of tobacco smoke, there are a number of *in vitro* models of toxicity using whole smoke exposure systems already in use [1–9]. Exposure system setups may differ greatly but their function and purpose is shared. A smoking machine/robot is used to generate and/or dilute mainstream whole smoke; this is delivered to an exposure chamber/module containing a simple or complex *in vitro* model of the lung at the air-liquid interface (ALI) so that a biological endpoint (usually related to one of the major smoking related diseases) can be assessed. As with the diversity of exposure systems and setups available, the same can be said of the *in vitro* model and endpoint testing. These may range from something as simple as a cytotoxicity assessment using a continuous cell line [1, 2, 8] through to complex endpoints assessing intracellular markers [9], or utilise more sophisticated cell cultures such as primary cell lines [10, 11], coculture systems, 3D-tissue models, or whole lung slices [3]. Currently, there are no defined *regulatory* protocols for whole smoke exposure systems, but these are being developed to support human *in vitro* models of disease. Aforementioned, there are a vast number of whole smoke exposure systems described in the literature, either commercially available [1–9] or one-off in-house setups [10, 12, 13]. Additionally, tobacco smoke is a concentrated and complex mix of at least 5,600 chemicals and toxicants found across two phases, the particulate (tar) and vapour phase [14]. Thus, assessing smoke dose is challenging. Consequently, when presenting whole smoke dose-response data, authors variously describe “dose” in many different ways: as a percentage of smoke; a fraction of smoke; ratios of smoke to air; puff number; total number of cigarettes smoked; total exposure of micrograms per culture insert; a flow rate of mixing air and vacuum applied to a smoke dilutor, all depending on the machine being used to generate and dilute the smoke [15]. Hence there is a need to accurately quantify particle and/or chemical deposition in our *in vitro* systems, such that comparisons of biological endpoints between different systems can be achieved with improved precision and accuracy both within and ultimately between laboratories. This is of increasing importance to scientists and regulators as it will allow consistent interpretation of results and quick cross-comparison of biological endpoints for defined smoke doses [16, 17]. Dosimetry (the assessment/measurement/quantification of smoke dose) is therefore a key element that can unite the biological effects of whole smoke generated by various and diverse exposure systems [3]. 

There are small numbers of chemicals or markers which can be quantified to assess tobacco smoke dosimetry; most of these dosimetry measurements assess the particulate phase due to the challenges of measuring individual components in the vapour phase, especially at higher smoke dilutions (lower concentrations) [15]. Chemicals/markers have been selected historically due to their facile quantification and include, but are not limited to: carbon monoxide [5] and oxides of nitrogen (NO_*x*_) which are in the gas phase; solanesol [18] and nicotine which are particulate markers; carbonyls which are split between phases; particulate matter as a whole either via gravimetric or chemical methods [7, 15]. 

**Figure 1:**
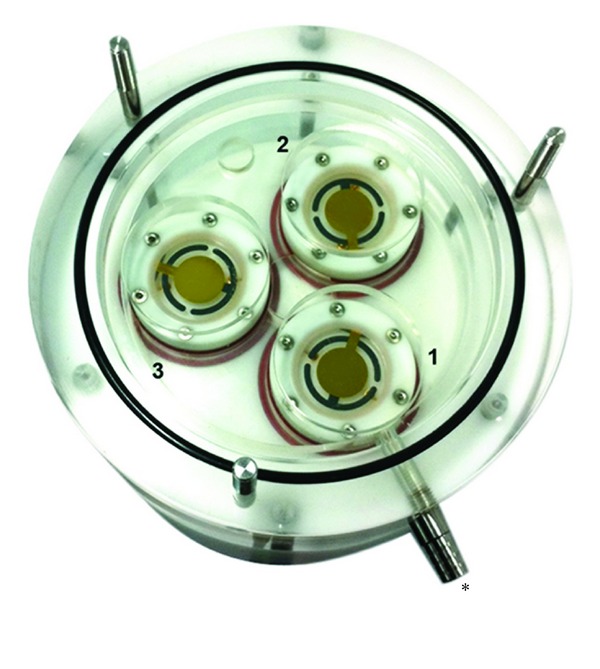
British American Tobacco's standard exposure chamber used for *in vitro* exposures to whole smoke at the air-liquid interface was modified to accommodate the 3 QCM units. The picture shows a top view of the 3-in-1 QCM exposure chamber base (crystal *ø* = 2.54 cm; cell support insert *ø* = 2.4 cm). QCM position 1 is proximal to the passive exhaust port ∗. QCM positions 2 and 3 are distal to the exhaust port and behind position 1 on the right and left, respectively.

Adamson et al. [15] reported a gravimetric method of particulate deposition quantification using a single quartz crystal microbalance (QCM) which robustly measured whole smoke particulate dose. The QCM is a sensitive gravimetric balance capable of measuring and detecting changes in mass of thin oscillating adherent films, within the nanogram range [19–22]. Previously, Adamson et al. [15] presented a new application (of an existing microbalance technology) of a single QCM unit in a chamber to assess the real-time deposition of tobacco smoke *in vitro* with verification by a chemical fluorescence method for smoke particle deposition. We now present a new, unique, and characterised dosimetry tool where the exposure chamber base accommodates 3 identical QCM units (Figure 1), termed the triplicate QCM chamber or *3-in-1* QCM chamber. Primarily, the study objectives were to assess/give an indication of regional deposition within the chamber (uniformity of particle deposition to cell cultures) where the previous single unit QCM could not show us this, and to increase robustness through the use of higher replicate numbers per run (from 1 to 3). For users of this exposure chamber, both sets of additional information are new and useful and could not have been provided by the single unit QCM. In this study we investigated the ability of the triplicate QCM to detect mass differences of whole smoke dilutions from 1 : 5 to 1 : 400 (smoke : air, volume : volume), for 30 minutes/run. The range 1 : 5–1 : 400 was selected as this represents the RM20S dilutions used in the laboratory to generate a biological dose response using robust *in vitro* primary and continuous cell cultures [1, 7, 9].

**Figure 2:**
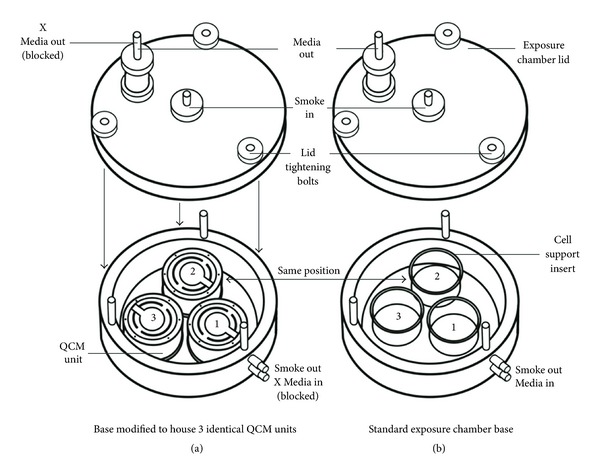
A schematic diagram of the triplicate QCM exposure chamber. (a) Crystals are installed directly into and replace the 3 positions where (b) cells would usually be exposed to whole smoke at the air-liquid interface. Illustration by J. Adamson.

Our results demonstrated that the QCM was able to discriminate mass between each dilution and was able to give information of regional deposition where ALI cell culture inserts would usually be at positions 1, 2, and 3 in the chamber (Figure 2). Furthermore, these results showed absolute agreement with the single unit QCM data previously reported [15]. Overall, the integrated triplicate QCM tool delivered robust, real-time, quantitative whole smoke mass measurements at nanogram levels and demonstrated an achievable dose response. 

## 2. Materials and Methods

### 2.1. Whole Cigarette Smoke Generation

Reference cigarettes (3R4F, 9.4 mg pack tar) (University of Kentucky, Lexington, KY, USA) were used for all experiments. Whole cigarette smoke was generated and diluted using two identical Borgwaldt RM20S smoking machines (Borgwaldt-kc, Hamburg, Germany) within the same laboratory, as previously described [1, 15]. The smoking machines and individual syringes from each machine were used interchangeably and at random and statistical analysis of the data thereafter showed that this had no effect on mass values obtained at the same dilution (data not shown). Five smoke dilutions were programmed as a ratio of smoke to air—1 : 5, 1 : 10, 1 : 25, 1 : 100, and 1 : 400 (smoke : air, volume : volume)—and 5 replicate experiments were conducted per dilution (*n* = 5). For all experiments, the machine smoked for 30 minute duration at ISO 4387 : 2000 smoking regime (35 mL puff over 2 seconds, once a minute, to a defined number of 6 puffs/cigarette). After the last cigarette was extinguished, the QCMs were left to record real-time data until all residual smoke in the chamber had deposited and mass values were observed to plateau and stabilise, usually taking an additional 10 minutes.

To prove deposition was due to smoke particulate alone, a Cambridge filter pad (CFP) (Borgwaldt-kc, Hamburg, Germany) was installed inline of smoke generation just prior to entry to the chamber for an additional single run of 1 hour (twice the duration of a standard run). As CFPs effectively trap 99.9% particulate matter [14, 23], anything detected by the QCM would therefore give an indication of nonparticulate mass activity within the exposure chamber during a smoke run.

### 2.2. The QCM Exposure Chamber Module

The previously described *in vitro* whole smoke exposure chamber manufactured for BAT by Curbridge Engineering (Southampton, UK) [7] had its base adapted to symmetrically house 3 identical commercially available QCM microbalance units (Vitrocell Systems GmbH, Waldkirch, Germany). The QCM housing units were installed with 5 MHz AT cut quartz crystals held between two Au/Cr polished electrodes, 1 inch (2.5 cm) diameter as described by Mülhopt et al. [19] (Figure 1). The QCM read at a resolution of 10 nanogram/cm^2^/second [15].

Before smoke exposure, the 3-in-1 QCM chamber was sealed and acclimatised in an incubator at 37°C to ensure quartz crystal stability. Quartz crystal stability was reached by zeroing the software before exposure until the baseline showed negligible drift (zero point stability) of less than 20 ng/cm^2^ over a duration of a few minutes. This took 5–10 minutes of zeroing prior to and between exposures in a stable environment. During longer periods of stability where the crystal was zeroed over a period of a few hours at constant environmental conditions, we were able to observe absolute zero point stability (0.00 ± 0.01 *μ*g/cm^2^) for 5 minutes prior to smoke exposure. During the deposition phase, the QCM recorded mass every 2 seconds for the 30 minute smoke exposure and 10 minute plateau phase, reporting as mass per unit area. Cell culture media was not included in the chamber for these mass measurements therefore media-in and media-out ports were blocked airtight to stop smoke leaking. After whole smoke exposure and between consecutive runs, quartz crystals were cleaned *in situ* (within the chamber, still screwed into their housing units). The crystal's surface was wiped with a soft lint-free tissue and 70% ethanol, and then polished to a shine. Crystals were not removed/replaced between readings; however, these should be replaced if broken or if their surface is severely scratched. Crystal stabilisation time was greatly reduced from up to 60 minutes to around 10 minutes when the crystals were cleaned *in situ*.

### 2.3. Statistics

**Figure 3:**
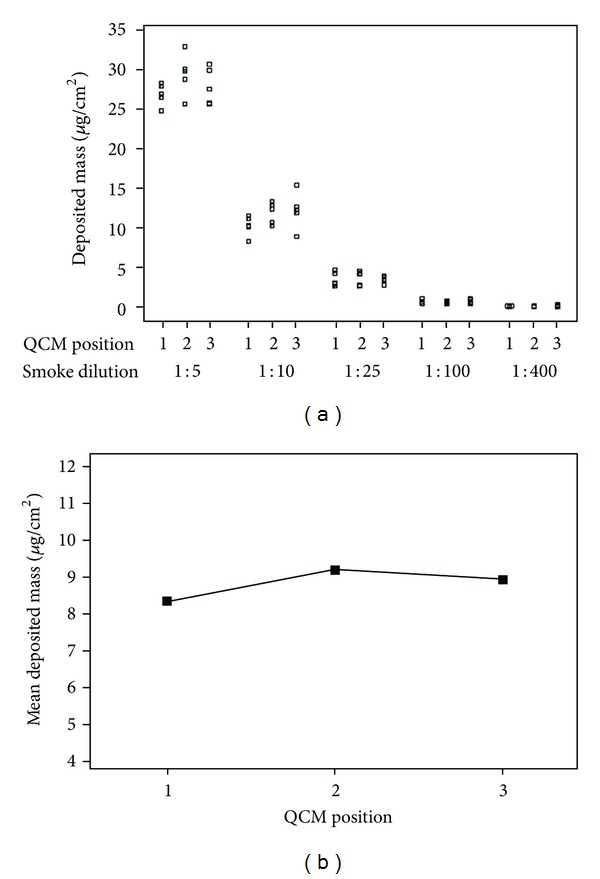
Triplicate QCM deposition data. (a) An individual value plot showing deposited particle mass quantification of ISO whole smoke (9.4 mg) diluted in the range 1 : 5–1 : 400 (smoke : air, v/v), *n* = 5/position. (b) A multi-vari chart of mean deposited mass at all five dilutions 1 : 5–1 : 400. The chart shows the average distribution of particulate deposition around the chamber within the range tested, from 25 values (5 dilutions at *n* = 5/dilution). There was no statistically significant difference in deposition between the three QCM positions.

**Figure 4:**
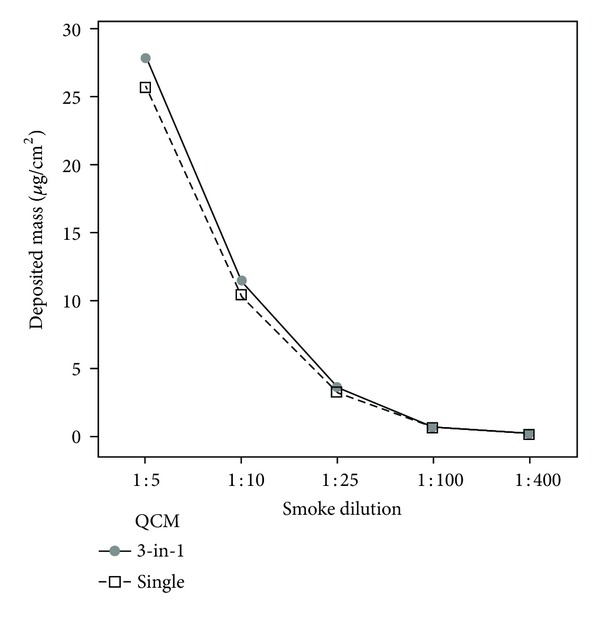
An interaction plot of the data means for the single unit QCM [15] and the 3-in-1 QCM, at the five airflows tested. There was no statistically significant difference between the two different devices.

**Figure 5:**
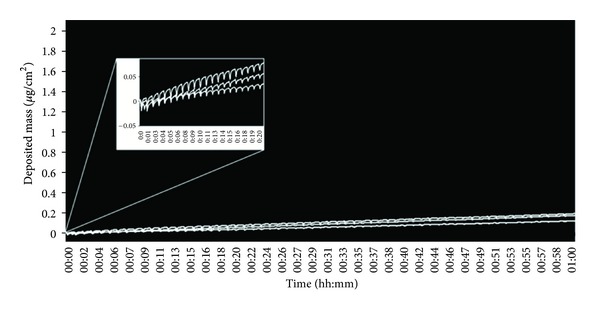
Deposition when a Cambridge filter pad (CFP) was placed prior to the QCM chamber to trap particulate matter. Cigarettes (3R4F) were smoked at the ISO regime at a dilution of 1 : 5 (smoke : air, v/v) for 1 hour. When the scale is adjusted to see the trace more clearly, note the distinct and repeated “n-shaped” pattern of mass increase and decrease per puff over time. The 3 traces represent the 3 QCMs within the chamber: QCM position 1, bottom; QCM position 2, top; QCM position 3, middle.

Data were reported as a mean ± standard deviation. The individual value plot of QCM-detected mass (Figure 3(a)), the multi-vari chart (Figure 3(b)), and the interaction plot comparing both QCM devices (Figure 4) were created using MINITAB v.16 statistical software. Main effects plots to check experimental variables (not shown) and a one-way ANOVA test using Tukey method to assess differences between QCM positions and QCM devices were also determined using MINITAB. All residual plots for all graphs generated by MINITAB were checked to ensure the quality of the data obtained. Real-time traces of deposited mass (Figure 5) were made using Microsoft Excel.

## 3. Results

The 3-in-1 QCM exposure chamber was able to record particulate mass in a dose-dependent manor in the dilution range 1 : 5–1 : 400 (smoke : air, v/v) (Figure 3(a)). Mean deposited mass ranged from 0.21 *μ*g/cm^2^ (210 ng/cm^2^) ±0.06 *μ*g/cm^2^ at the most dilute dose of 1 : 400, up to 28.00 *μ*g/cm^2^ (27,998 ng/cm^2^) ±2.25 *μ*g/cm^2^ at the most concentrated dose of 1 : 5. This mass range was obtained from the means of all three QCM positions per dose (*n* = 5/position). A one-way analysis of variance (ANOVA) test showed that, as expected, there were significant differences between dilutions (*P* ≤ 0.05); however, 1 : 100 and 1 : 400 dilutions had grouped confidence intervals indicating that overall mass values were in the same range at these high dilutions.

The data were presented by individual QCM position so that the regional deposition around the chamber could be assessed (Figure 3(a)). QCM position 1 is most proximal to the passive exhaust port where smoke exits the chamber, whereas positions 2 and 3 are paired distal to the port, right and left, respectively (Figures 1 and 2). To assess the distribution of particulate deposition around the chamber positions *at all the dilutions tested*, a multi-vari chart was produced (Figure 3(b)). This chart demonstrates the uniformity of particle deposition in the biological exposure range, showing the mean deposited mass of the three QCM positions at all five dilutions tested. There were no significant differences between QCM positions 1, 2, and 3 for all dilutions tested—1 : 5 (*P* = 0.205), 1 : 10 (*P* = 0.186), 1 : 25 (*P* = 0.923), 1 : 100 (*P* = 0.794), and 1 : 400 (*P* = 0.435). 

The dose-response data generated from this triplicate QCM tool was compared with the aforementioned single unit QCM [15], and good parity was observed between them with no statistically significant difference between the different tools at all dilutions tested (Figure 4). For dilutions 1 : 10–1 : 400 deposition was highly comparable between the two tools (≤5%) (Table 1). At the most concentrated dilution of 1 : 5, the difference in detected deposited mass between the tools was 8.4% (Table 1). Considering the entire range tested, the difference between both tools at all dilutions was <10% which we would consider to be an acceptable difference (fit for purpose).

Finally, a Cambridge filter pad (CFP) was placed inline of smoke generation to occlude particulate entering the chamber—the purpose was to assess semivolatile deposition or potentially the behavior of other gases. Figure 5 shows a real-time trace of particulate detection during an extended smoke exposure of 1 hour at the highest smoke concentration of 1 : 5 (smoke : air, v/v) with a CFP inline. The trace showed that mass increased over time, but this increase was nominal considering the high tar level of 3R4F cigarettes (9.4 mg) and the high concentration of whole smoke chosen (1 : 5). There was an increase of 78 ng/cm^2^ with the CFP inline compared with particulate matter deposition of 27,998 ng/cm^2^ for diluted whole smoke at the same dilution of 1 : 5. Furthermore, after 1 hour of smoking the overall mass reached was <200 ng/cm^2^ (mean of the 3 positions was 156 ng/cm^2^). Whilst the initial chart would suggest that negligible mass was detected and that the traces were flat lined, however, when the scale was adjusted (Figure 5 pullout) a repeated and conserved saw-tooth pattern was observed, indicative (at least in time) of some transient mass deposition, probably constituents of diluted vapour phase or water vapour entering the chamber. In addition, a further inline CFP measurement at the higher smoke dilution of 1 : 400 showed a similar saw-tooth mass deposition pattern of negligible cumulative mass (not reported). The single “n-shaped” unit observed here would suggest a small increase, plateau, and decrease in mass as diluted vapour phase smoke was delivered to the chamber. This could be an indication of semivolatile deposition on the QCM surface followed by evaporation as airflow through the chamber changed per puff.

As noted, cumulative mass over 60 minutes with the CFP inline was 156 ng/cm^2^/hr. If the sum of the transient peaks is calculated with subsequent reevaporation ignored (Figure 5), the cumulative mass was approximately 620 ng/cm^2^/hr. This amount of detected mass is too low to be water vapour or vapour phase in its totality. The repeat pattern does suggest some components of the vapour phase but it would be difficult to identify semivapour phase revolatilising here without additional chemistry analysis. Rodgman and Perfetti [24] cite an example cigarette of generating 7.5 mg vapour phase and 17.4 mg NFDPM (nicotine free dry particulate matter). If this proportionality is maintained for the 3R4F cigarette, ISO yields of 9.4 mg NFDPM, 0.87 mg water (measured), and 4.1 mg vapour phase (estimated) and calculating orders of magnitude and the contribution of particulate, vapour, and water from a 3R4F cigarette, we would expect to see about 14,000 ng for total vapour phase and about 2,800 ng for water. Thus what we have identified on the QCM with the particulate phase occluded is too low to be either water or total vapour.

## 4. Discussion

In this study we present a new and unique tool (Figure 1) to quantify diluted whole smoke particulate matter deposition *in vitro*. This tool measured real-time deposition at a range of dilutions 1 : 5–1 : 400 (smoke : air, v/v) resulting in quantification of deposited particulate matter in the range 0.21–28.00 *μ*g/cm^2^, most dilute to most concentrated smoke dilution (Figure 3(a)). The technology was able to detect deposited mass with such resolution that it could identify puff-by-puff profiles, probably of some vapour phase constituents alone (when particles were occluded with a filter pad) (Figure 5). For the first time, mass values were read in triplicate by 3 identical QCM units installed into the exposure chamber, each in exactly the same location as the cell culture inserts would be exposed to whole smoke at the ALI (Figure 2). This information is particularly useful to users of the chamber as it demonstrates uniformity, with no difference in particle deposition to the 3 positions where culture inserts would be exposed to smoke. 

To further assess the sensitivity of the QCM technology, a Cambridge filter pad (CFP) was placed inline of smoke generation to effectively occlude the particulate fraction of whole smoke. The hypothesis here was that no particulate mass would be detected and anything being detected would be derived from the diluted gas/vapour phase delivered to the chamber. As predicted, nominal mass was detected in the range <200 ng/cm^2^ in 1 hour; however, a distinct and repeated saw-tooth pattern was observed in the real-time trace (Figure 5). Although we are yet to identify what is being deposited on the QCM surface when the CFP is inline, we predict this could be an indication of some vapour phase components condensing and evaporating as airflow through the chamber changes per puff. Elution, chemical analysis, and quantification will help us potentially identify what part of the vapour phase is deposited on the exposed QCM surface, and we plan to do this in subsequent studies.

There are unique challenges not only in the identification of smoke components but also their quantitation [14]. Of the particulate matter which is being quantified by the QCM, grossly it approximates 16% water, 6% nicotine, and 78% “tar” (NFDPM), of which the major components of NFDPM are alcohols (20%), acids (17%), and aldehydes and ketones (14%) [14]. Sophisticated analytical tools, such as gas-chromatography mass spectrometry (GC/MS), are required to further identify these compounds. The challenge persists when it is estimated that components representing less than 1 mg of the particulate phase remain unidentified, averaging in the low nanogram and picogram levels [14]. At the ALI cell exposure surface, the particles depositing will have a soluble and an insoluble fraction. Therefore there will be species available to the thin surface liquid which lines the cell monolayer. There is little direct information published on soluble particle components and cellular interactions at the ALI, but recent in-house data suggests that a soluble fraction is 40%–90% of the particle droplet. Lastly and as previously discussed, vapour phase components are largely overlooked by this sampling method; Figure 5 demonstrates the minor quantities of material being deposited when the diluted smoke passes through a CFP.

We previously published a study demonstrating the use of a single unit QCM in the same exposure chamber [15]. Based on that data obtained, and usefulness of the tool, we further developed the triplicate chamber to support *in vitro* testing and confirm uniformity of particle deposition around the chamber, where the single unit QCM device could not show us this. As well as allowing us to assess potential positional deposition around the exposure chamber, this expanded tool allowed us to increase dosimetry replicate number per exposure. Within each dilution tested, there were no statistically significant differences in deposition around the 3 positions in the chamber (Figure 3(b)) with *P* values for all dilutions greater than 0.1. 

Data obtained from the triplicate QCM exposure chamber compared to that obtained from the previously published single unit device [15] confirmed they were performing equally (Figure 4). At dilutions greater than 1 : 10, the results obtained from the two tools differed by 5% or less in terms of quantified mean deposited mass; this is to be expected as interrun variability. At 1 : 5 dilution, the difference was slightly higher at 8.4% (Table 1). But overall, these differences were all <10% (a range of 6.1% across the dilution range tested) which we would deem to be acceptable.

We have presented a new study demonstrating an additional/expanded, novel, and simple tool used to quantify tobacco smoke deposition *in vitro* in real time. The QCM technology itself is not new and in the past it has been used for such activities as environmental monitoring [21, 25], biological applications [26, 27], and inhalation toxicity assessment of (nontobacco) aerosols and engineered nanoparticles *in vitro* [19, 28, 29]. However, the utilisation and combination of these technologies for assessment of whole smoke exposure *in vitro* is novel. Certainly, QCM technology has many potential applications in the field of inhalation toxicology, not just tobacco smoke.

As discussed previously, there are many different setups available to industry and other respiratory toxicology research groups; in this study we used the Borgwaldt RM20S to dilute and deliver mainstream smoke to the triplicate QCM. Because so many different smoking machine and exposure systems are used, it is clear there needs to be a simple and aligned method of measuring dose *in vitro*; this is especially the case with the current switch from liquid to ALI exposures of aerosols *in vitro* [16]. Dosimetry is therefore a key element that can link the biological effects of tobacco smoke generated by various exposure systems [3]. As dose measurements with a QCM allow real-time, fast, and accurate determination of the cell deposited dose [28], we believe microbalance technology offers a way forward, not only for the assessment of whole smoke but also for other inhalable toxicants [30]. In terms of human smokers, this tool can be used to generate deposition values *in vitro* consistent with values measured in human smokers. For example, in this study QCMs detected deposition of diluted particulate matter *in vitro* in the range 0.21–28.00 *μ*g/cm^2^. For context, McAughey et al. [31] estimated daily deposited lung doses in the order of 40–100 *μ*g/cm^2^ in the extrathoracic region, 1.0–2.0 *μ*g/cm^2^ in the bronchial/bronchiolar region, and 0.1–0.2 *μ*g/cm^2^ in the alveolar-interstitial region, based on human smoking data for lung retention and regional deposition. These human values in the lower range 0.1–2.0 *μ*g/cm^2^ (bronchial-alveolar) further align with our *in vitro* exposure system: primarily it is cells of the bronchial epithelium or alveolar (A549) cells which would be used in these biological models of disease and exposed at the ALI in this chamber.

The 3-in-1 tool described here was compared to the previously published single QCM chamber (Figure 4). Currently, neither of the these QCM chambers can accommodate cell cultures at the same time as deposition quantification (the 3-in-1 cannot house cells as there is no space, and yet the single QCM has not been tested concurrently with media and/or cell cultures). However, this is something we will look at in future studies. Thus the operational advantage of the triplicate QCM versus the single QCM chamber is to increase replicate number from one to three during a single smoke exposure. This would be useful for deposition dose-range studies of different cigarettes types, for example. To obtain directly comparable chemistry deposition data alongside QCM deposition, the single QCM chamber should be used as positions 1 and 3 are available to house nude cell support inserts. Furthermore, if cell cultures could be supported on inserts for a whole smoke exposure without basal media, then this single unit tool could be used to obtain deposition data at the same time as a biological response. As the data have shown here, there is no statistically significant difference between the tools, thus both QCM chambers have applicability depending on the design of the experiment and the desired outcome.

## 5. Conclusion

In this study, the QCM successfully determined deposited smoke mass generated from the Borgwaldt RM20S Smoking Machine, but it can easily be adapted to assess smoke mass produced from other commercially available smoking machines. Consequently, we predict that this tool may help align smoke exposure technologies. Additionally, the 3-in-1 QCM exposure chamber, although designed to quantify deposited smoke mass *in vitro*, could potentially be used to assess other types of aerosol delivery to *in vitro* cultures, such as manufactured particles and fibres, some aerosolised cosmetics, household products, or pesticides. Certainly aerosols similar to tobacco whole smoke which are submicron liquid spherical droplets would be easy to detect. Hence the scope of this tool is vast. The 3-in-1 QCM also offers a robust and efficient alternative to traditional chemistry methods or supports such methods and delivers additional benefits of particulate quantification: (1) it is single person operated; (2) no other resource or reagents are required; (3) data is generated fast, in real time and because of this; (4) it can be used as a QC device to rapidly assess the status of a smoke exposure or identify issues with machine smoking and dilution.

Until now, there have been few (if any) reliable and accurate methods of determining particulate dose delivered to the exposure chamber in real time. Thus biological dose-response data has been presented as the machine's programmed ratio of smoke to air, with the expectation that smoke dilutions delivered from the smoking robot are robust and repeatable. In the case of the Borgwaldt RM20S, precision of smoke dilution and delivery has been previously investigated and proven to be reliable by a number of physicochemical and analytical methods, and this has been published [1, 5]. The addition of this QCM work further supports our confidence in the machine's ability to dilute and deliver smoke reliably.

Finally, we propose to continue to use these QCM tools to support the preclinical *in vitro* evaluation of tobacco products and accurately quantify particle dose delivery to cell cultures. In addition, this tool has been shown to align generated deposition values *in vitro* consistent with values measured in human smokers. 

## Figures and Tables

**Table 1:** Mean deposited mass values obtained from the single unit QCM [15] (*n* = 5) compared to the 3-in-1 QCM unit (*n* = 15); the percentage difference between mean mass detected by the two tools; the *P* values indicating no significant difference between tools at all dilutions tested. For the single unit, the values were the mean of 5 repeat experiments with the QCM (in position 2 of the chamber) [15]; for the triplicate QCM the values were the mean of 5 repeat experiments and 3 QCM positions per repeat (*n* = 15).

	Mean deposited mass (*μ*g/cm^2^) ± SD				
Dilution (1 : *X*)	5	10	25	100	400
Single unit	25.75 ± 2.30	10.51 ± 0.42	3.29 ± 0.24	0.69 ± 0.09	0.22 ± 0.03
Triplicate unit	28.00 ± 2.25	11.51 ± 1.81	3.64 ± 0.72	0.75 ± 0.20	0.21 ± 0.06
Difference (%)	8.4	4.5	5.2	4.9	2.3
*P* value	0.071	0.245	0.304	0.503	0.573

## References

[1] Adamson J, Azzopardi D, Errington G, Dickens C, McAughey J, Gaca MD (2011). Assessment of an *in vitro* whole cigarette smoke exposure system: the Borgwaldt RM20S 8-syringe smoking machine. *Chemistry Central Journal*.

[2] Aufderheide M, Knebel JW, Ritter D (2003). An improved *in vitro* model for testing the pulmonary toxicity of complex mixtures such as cigarette smoke. *Experimental and Toxicologic Pathology*.

[3] Chi-Jen Lin J, Jean-Phillippe R, Verreault J (2012). An *ex vivo* approach to the differential parenchymal responses induced by cigarette whole smoke and its vapor phase. *Toxicology*.

[4] Fukano Y, Ogura M, Eguchi K, Shibagaki M, Suzuki M (2004). Modified procedure of a direct *in vitro* exposure system for mammalian cells to whole cigarette smoke. *Experimental and Toxicologic Pathology*.

[5] Kaur N, Lacasse M, Roy JP (2010). Evaluation of precision and accuracy of the Borgwaldt RM20S® smoking machine designed for *in vitro* exposure. *Inhalation Toxicology*.

[6] Maunders H, Patwardhan S, Phillips J, Clack A, Richter A (2007). Human bronchial epithelial cell transcriptome: gene expression changes following acute exposure to whole cigarette smoke *in vitro*. *American Journal of Physiology*.

[7] Phillips J, Kluss B, Richter A, Massey ED (2005). Exposure of bronchial epithelial cells to whole cigarette smoke: assessment of cellular responses. *ATLA Alternatives to Laboratory Animals*.

[8] Scian MJ, Oldham MJ, Kane DB, Edmiston JS, McKinney WJ (2009). Characterization of a whole smoke *in vitro* exposure system (Burghart Mimic Smoker-01). *Inhalation Toxicology*.

[9] Thorne D, Wilson J, Kumaravel TS, Massey ED, McEwan M (2009). Measurement of oxidative DNA damage induced by mainstream cigarette smoke in cultured NCI-H292 human pulmonary carcinoma cells. *Mutation Research*.

[10] Zhang W, Case S, Bowler RP, Martin RJ, Jiang DI, Chu HW (2011). Cigarette smoke modulates PGE2 and host defence against Moraxella catarrhalis infection in human airway epithelial cells. *Respirology*.

[11] Beisswenger C, Platz J, Seifart C, Vogelmeier C, Bals R (2004). Exposure of differentiated airway epithelial cells to volatile smoke *in vitro*. *Respiration*.

[12] St-Laurent J, Proulx LI, Boulet LP, Bissonnette E (2009). Comparison of two *in vitro* models of cigarette smoke exposure. *Inhalation Toxicology*.

[13] Gualerzi A, Sciarabba M, Tartaglia G, Sforza C, Donetti E (2012). Acute effects of cigarette smoke on three-dimensional cultures of normal human oral mucosa. *Inhalation Toxicology*.

[14] Perfetti TA, Rodgman A (2011). The complexity of tobacco and tobacco smoke. *Beitrage zur Tabakforschung International*.

[15] Adamson J, Hughes S, Azzopardi D, McAughey J, Gaca M (2012). Real-time assessment of cigarette smoke particle deposition *in vitro*. *Chemistry Central Journal*.

[16] Paur HR, Cassee FR, Teeguarden J (2011). *In-vitro* cell exposure studies for the assessment of nanoparticle toxicity in the lung-A dialog between aerosol science and biology. *Journal of Aerosol Science*.

[17] Teeguarden JG, Hinderliter PM, Orr G, Thrall BD, Pounds JG (2007). Particokinetics *in vitro*: dosimetry considerations for *in vitro* nanoparticle toxicity assessments. *Toxicological Sciences*.

[18] Armitage AK, Dixon M, Frost BE, Mariner DC, Sinclair NM (2004). The effect of inhalation volume and breath-hold duration on the retention of nicotine and solanesol in the human respiratory tract and on subsequent plasma nicotine concentrations during cigarette smoking. *Beiträge zur Tabakforschung International*.

[19] Mülhopt S, Diabaté S, Krebs T, Weiss C, Paur HR (2009). Lung toxicity determination by *in vitro* exposure at the air liquid interface with an integrated online dose measurement. *Journal of Physics: Conference Series*.

[20] Smith AL, Shirazi HM (2005). Principles of quartz crystal microbalance/heat conduction calorimetry: measurement of the sorption enthalpy of hydrogen in palladium. *Thermochimica Acta*.

[21] Yuwono A, Schulze Lammers P (2004). Odour pollution in the environment and the detection instrumentation. *Agricultural Engineering International*.

[22] Saubrey G (1959). Verwendung von Schwingquarzen zur Wagung dunner Schichten und zur Mikrowagung. *Zeitschrift für Physik*.

[23] Johnson MD, Schilz J, Djordjevic MV, Rice JR, Shields PG (2009). Evaluation of *in vitro* assays for assessing the toxicity of cigarette smoke and smokeless tobacco. *Cancer Epidemiology Biomarkers and Prevention*.

[24] Rodgman A, Perfetti TA (2009). *The Chemical Components of Tobacco and Tobacco Smoke*.

[25] Klepeis NE, Ott WR, Switzer P (2007). Real-time measurement of outdoor tobacco smoke particles. *Journal of the Air and Waste Management Association*.

[26] Uttenthaler E, Schräml M, Mandel J, Drost S (2001). Ultrasensitive quartz crystal microbalance sensors for detection of M13-Phages in liquids. *Biosensors and Bioelectronics*.

[27] Yeh HC, Turner RS, Jones RK, Muggenburg BA, Lundgren DL, Smith JP (1995). Characterization of aerosols produced during surgical procedures in hospitals. *Aerosol Science and Technology*.

[28] Lenz GA, Karg E, Lentner B (2009). A dose-controlled system for air-liquid interface cell exposure and application to zinc oxide nanoparticles. *Particle and Fibre Toxicology*.

[29] Müller L, Gasser M, O’Raemy D (2011). Realistic exposure methods for investigating the interaction of nanoparticles with the lung at the air-liquid interface *in vitro*. *Insciences Journal*.

[30] Bakand S, Hayes A (2010). Troubleshooting methods for toxicity testing of airborne chemicals *in vitro*. *Journal of Pharmacological and Toxicological Methods*.

[31] McAughey JJ, McGrath CJ, Dickens CJ (2009). Particle metrics for mainstream tobacco smoke: implications for dose. *Journal of Aerosol Medicine & Pulmonary Drug Delivery*.

